# Sinomenine Hydrochloride Protects against Polymicrobial Sepsis via Autophagy

**DOI:** 10.3390/ijms16022559

**Published:** 2015-01-23

**Authors:** Yu Jiang, Min Gao, Wenmei Wang, Yuejiao Lang, Zhongyi Tong, Kangkai Wang, Huali Zhang, Guangwen Chen, Meidong Liu, Yongming Yao, Xianzhong Xiao

**Affiliations:** 1 Laboratory of Shock, Department of Pathophysiology, Xiangya School of Medicine, Central South University, Changsha 410008, China; E-Mails: jiangyu146@hotmail.com (Y.J.); wwmyouc@163.com (W.W.); langyj386@sina.com (Y.L.); tongzhongyi2008@163.com (Z.T.); wangkangkai@csu.edu.cn (K.W.); Zhanghuali@csu.edu.cn (H.Z.); Chen02gw@yahoo.com (G.C.); LiuMD2005@126.com (M.L.); 2 Department of Critical Care Medicine, the Third Xiangya Hospital, Central South University, Changsha 410013, China; E-Mail: gaomin1984@aliyun.com; 3 Department of Microbiology and Immunology, Burns Institute, First Hospital Affiliated to the Chinese PLA General Hospital, Beijing 100037, China; E-Mail: c_ff@sina.com

**Keywords:** sepsis, cecal ligation and puncture (CLP), sinomenine hydrochloride (SIN-HCl), autophagy, peritoneal macrophages (PM)

## Abstract

Sepsis, a systemic inflammatory response to infection, is the major cause of death in intensive care units (ICUs). The mortality rate of sepsis remains high even though the treatment and understanding of sepsis both continue to improve. Sinomenine (SIN) is a natural alkaloid extracted from Chinese medicinal plant *Sinomenium acutum*, and its hydrochloride salt (Sinomenine hydrochloride, SIN-HCl) is widely used to treat rheumatoid arthritis (RA). However, its role in sepsis remains unclear. In the present study, we investigated the role of SIN-HCl in sepsis induced by cecal ligation and puncture (CLP) in BALB/c mice and the corresponding mechanism. SIN-HCl treatment improved the survival of BALB/c mice that were subjected to CLP and reduced multiple organ dysfunction and the release of systemic inflammatory mediators. Autophagy activities were examined using Western blotting. The results showed that CLP-induced autophagy was elevated, and SIN-HCl treatment further strengthened the autophagy activity. Autophagy blocker 3-methyladenine (3-MA) was used to investigate the mechanism of SIN-HCl *in vitro*. Autophagy activities were determined by examining the autophagosome formation, which was shown as microtubule-associated protein light chain 3 (LC3) puncta with green immunofluorescence. SIN-HCl reduced lipopolysaccharide (LPS)-induced inflammatory cytokine release and increased autophagy in peritoneal macrophages (PM). 3-MA significantly decreased autophagosome formation induced by LPS and SIN-HCl. The decrease of inflammatory cytokines caused by SIN-HCl was partially aggravated by 3-MA treatment. Taken together, our results indicated that SIN-HCl could improve survival, reduce organ damage, and attenuate the release of inflammatory cytokines induced by CLP, at least in part through regulating autophagy activities.

## 1. Introduction

Sepsis, a systemic inflammatory response to infection, is the major cause of death in ICUs. Sepsis-induced chaotic processes in the host immune system cause tissue injury, organ dysfunction, and subsequent organ failure [1]. The mortality rate of sepsis remains high even though the treatment and understanding of sepsis continue to improve [2]. Therefore, it is urgent to understand the mechanisms underlying the pathogenesis of sepsis and develop new therapeutic strategies.

Autophagy is a bulk intracelluar degradation system that delivers cytoplasmic proteins and organelles to lysosomes for degradation and recycling [3]. Under normal conditions, autophagy plays important roles in development, cell survival and differentiation [4,5]. However, it may also participate in pathological processes. Many studies have reported that autophagy is induced in patients with sepsis or CLP animal model. For instance, autophagic vacuolization in hepatocytes, which can be identified by electron microscopy, was observed in patients with sepsis [6]. Autophagy was also induced in multiple organs, including the heart, lungs, liver, and kidneys, in the CLP model [7,8,9,10,11,12]. Nevertheless, whether the process of autophagy is generally beneficial or harmful to the immune defense and other cell functions during sepsis is still not well defined.

**Figure 1:**
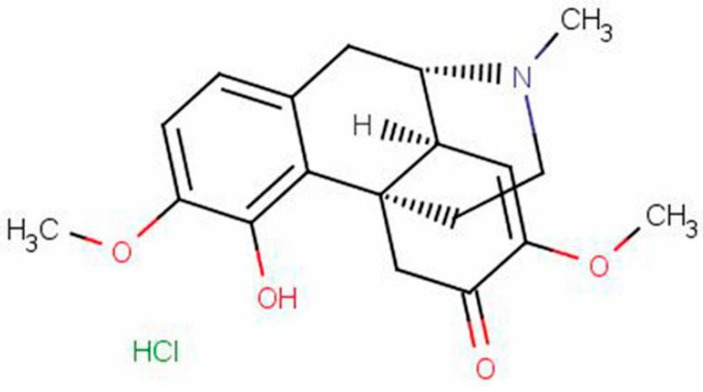
The structure of sinomenine hydrochloride (SIN-HCl).

SIN [(9α,13α,14α)-7,8-didehydro-4-hydroxy-3,7-dimethoxy-17-methyl-morphinane-6-one] is a natural alkaloid extracted from Chinese medicinal plant *Sinomenium acutum*, and its hydrochloride salt, SIN-HCl (Figure 1) is widely used as an immune regulatory drug in rheumatoid arthritis (RA) treatment [13,14]. Numerous pharmacological and clinical studies performed in China and Japan demonstrated that SIN possessed anti-inflammatory [15], immunoregulatory [16], and anti-angiogenic [17] properties. As systemic inflammatory response and immunosuppression exist simultaneously in sepsis, we hypothesized that SIN-HCl might be involved in anti-inflammation and immunoregulation in CLP-induced sepsis. However, the exact effects and mechanisms remain unclear.

We explain here that SIN-HCl had a protective effect against CLP-induced sepsis. It increased the survival of mice, reduced organ damage, and attenuated the release of inflammatory cytokines from systemic autophagy induction. Taken together, these data strongly suggested that SIN-HCl might present a new therapeutic option for sepsis through modulation of autophagy.

## 2. Results

### 2.1. SIN-HCl Protected against Polymicrobial Sepsis in Model Mice and Attenuated Multiple Organ Dysfunction and Systemic Inflammatory Response

**Figure 2:**
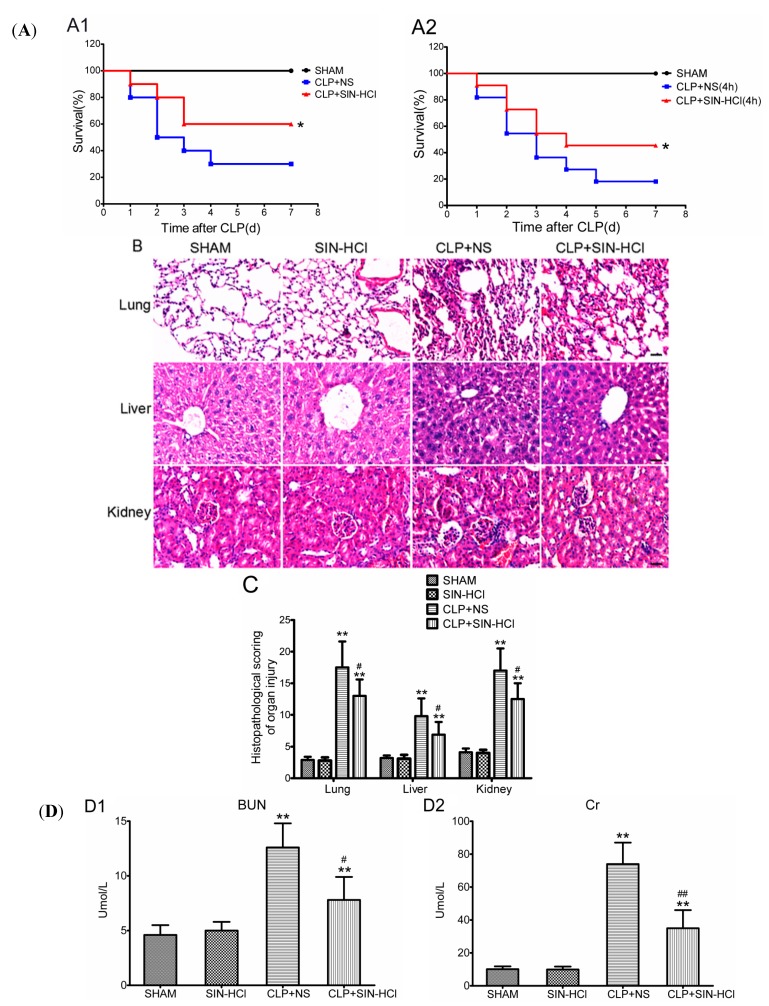

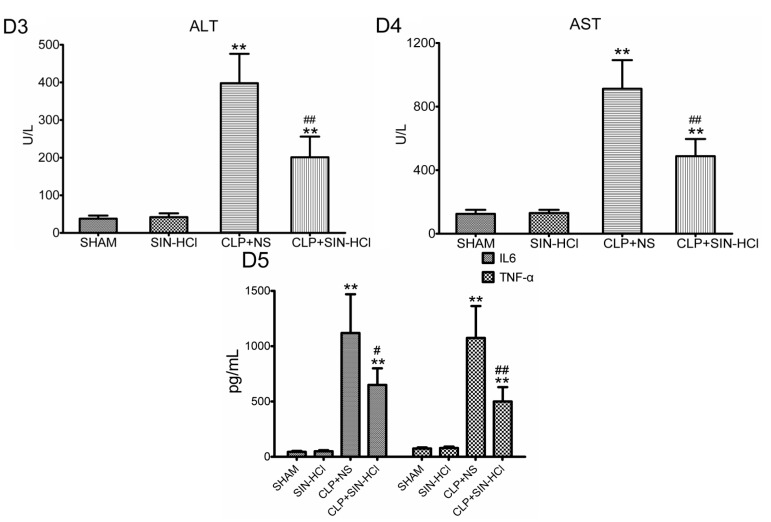
SIN-HCl protected a mouse model against polymicrobial sepsis and attenuated the multiple organ dysfunction and systemic inflammatory response. (**A**) SIN-HCl improved the survival rate of mice with CLP-induced sepsis. BALB/c mice were subjected to CLP surgery. SIN-HCl (100 mg/kg) was administered by hypodermic injection at 0 h (**A1**) or 4 h (**A2**) after CLP surgery. NS was used as the control. * *p* < 0.05 when compared with CLP + NS group. Data were pooled from three experiments, *n* = 10 per group in each experiment; (**B**) The lungs, liver, and kidneys were stained with HE. The organ damage induced by CLP was attenuated significantly in SIN-HCl-treated BALB/c mice, as evidenced by less inflammatory cell infiltration, reduced exudate blockage of capillary, and less substantial interstitial cellular degeneration and necrosis in the lungs, liver, and kidneys. Scale bar was equivalent to 50 μm. Images were the selected representatives of each group, which had six to eight mice; (**C**) Semi-quantitative analysis of lung, liver, and kidney injury. ** *p* < 0.01 when compared with sham BALB/c mice, *n* ≥ 6; ^#^ *p* < 0.05 when compared with the NS-treated BALB/c mice, *n* ≥ 6; and (**D**) Biochemical measurements of the serum (**D1–D4**) were performed on automatic biochemical analyzer, and concentrations of serum IL-6 and TNF-α (**D5**) were determined by ELISA. ** *p* < 0.01 when compared with sham BALB/c mice, *n* ≥ 6; ^#^ *p* < 0.05 and ^##^ *p* < 0.01 when compared with the CLP and NS-treated BALB/c mice, *n* ≥ 6.

CLP appears to be a reliable and clinically relevant animal model of the human septic conditions because the procedure produces an endogenous polymicrobial infection, which could mimic peritonitis and sepsis in clinic [18]. We determined the effect of SIN-HCl on the survival rate of BALB/c mice that were subjected to a CLP procedure. Immediate administration and post-administration of SIN-HCl 100 mg/kg, hypodermic injection (i.h.) both revealed significantly reduced mortality when compared with normal saline (NS)-treated controls (Figure 2A). Moreover, we determined the role of SIN-HCl in CLP-induced organ damages. As shown in Figure 2B, the organ damage induced by CLP was attenuated significantly in SIN-HCl-treated mice, as evidenced by less inflammatory cell infiltration, reduced exudate blockage of capillary, and lower levels of interstitial cellular degeneration and necrosis, in the lungs, liver, and kidneys. The HE staining was analyzed and showed in Figure 2C. The variation of serum biochemical parameters at least in part reflected the severity of organ damage. CLP operation caused the increased blood urine nitrogen (BUN), creatinine (Cr), alanine transaminase (ALT), and aspartate Transaminase (AST), which were antagonized by SIN-HCl treatment (Figure 2D). Increases in systemic inflammatory cytokines, such as IL-6 and TNF-α, are biomarkers for and causes of poor prognosis in sepsis [19]. We measured the serum inflammatory cytokines using ELISA. The data showed that serum IL-6 and TNF-α levels increased significantly in BALB/c mice after being subjected to CLP, and SIN-HCl treatment lowered their levels in serum (Figure 2D). These results indicated that CLP-induced polymicrobial sepsis could cause severe organ dysfunction and systemic inflammatory response; however, SIN-HCl treatment attenuated this damage and played a protective role in the CLP model.

### 2.2. CLP-Induced Autophagy in Multiple Organs Was Strengthened in SIN-HCl Treatment in Mice

**Figure 3:**
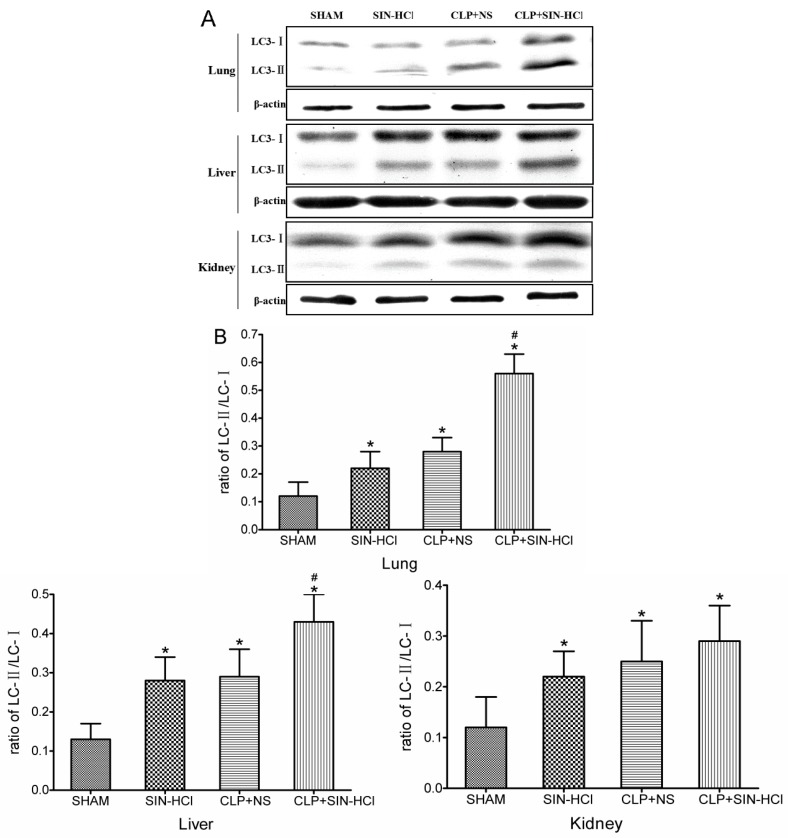
CLP-induced autophagy in multiple organs was strengthened in SIN-HCl-treated mice. (**A**) SIN-HCl treatment strengthened CLP-induced autophagy in multiple organs of the mice. The protein level of LC3 was measured using an anti-LC3 antibody. The ratios of LC3-II:LC3-I in the lungs, liver, and kidneys were higher at 12 h after CLP procedure or SIN-HCl treatment. SIN-HCl further increased the ratios of LC3-II:LC3-I in the lungs and livers of mice that had undergone the CLP; (**B**) Western blotting was quantitatively analyzed, * *p* < 0.05 when compared with sham BALB/c mice, *n* ≥ 5; ^#^ *p* < 0.05 when compared with the CLP + NS-treated BALB/c mice, *n* ≥ 5.

Autophagy was induced by various types of stresses. Previous studies had demonstrated that sepsis or LPS could induce autophagy. Our earlier study found that SIN-HCl could also induce autophagy in the RAW264.7 cell line (Figure S1); therefore, we hypothesized that autophagy might be involved in the protective effects of SIN-HCl in the CLP model. First, we confirmed that CLP operation could induce transformation of autophagic protein LC3-I to LC3-II in various mouse tissues and the SIN-HCl treatment could activate autophagy in those mouse tissues *in vivo* (Figure 3). Next, we evaluated whether SIN-HCl could modulate autophagy in the context of the CLP model. We found that the ratio of LC3-II:LC3-I in the lungs and liver was further increased after SIN-HCl treatment in comparison with the mice that only underwent CLP (Figure 3).

### 2.3. SIN-HCl Blocked Lps-Induced Inflammatory Cytokine Release Induced by LPS and Increased Autophagy in Peritoneal Macrophages (PM)

**Figure 4:**
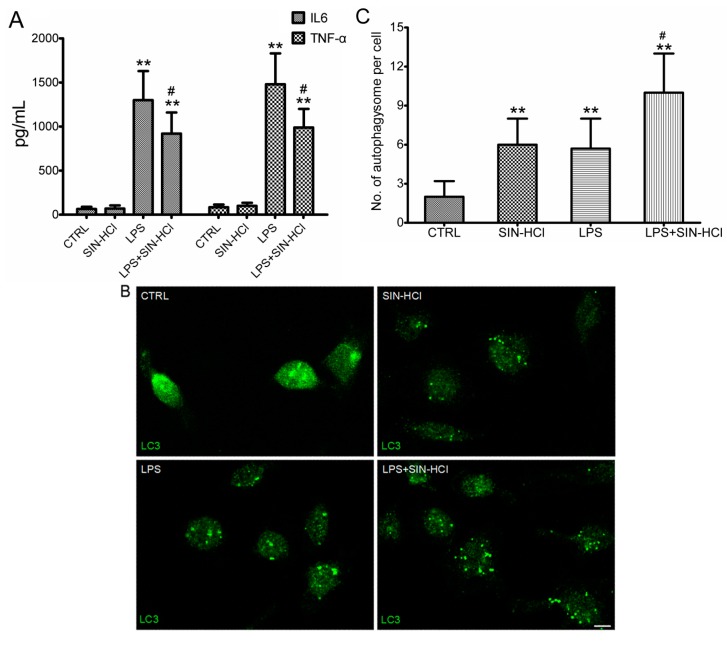
SIN-HCl blocked LPS-induced inflammatory cytokine release and increased autophagy in peritoneal macrophages (PM). (**A**) Primary PM were cultured for 12 h with LPS (100 ng/mL), SIN-HCl (100 mM), or a combination of these two reagents (as indicated). The levels of IL-6 and TNF-α in culture media were measured. ** *p* < 0.01 when compared with PBS group, *n* ≥ 6; ^#^ *p* < 0.05 when compared with the LPS group, *n* ≥ 6; (**B**) Primary PM were treated with LPS (100 ng/mL), SIN-HCl (100 mM), or a combination of these two reagents (as indicated) for 12 h before immunofluorescent detection of LC3 protein using an anti-LC3 antibody, Scale bar = 10 μm and (**C**) Autophagosomes per cell in PM were counted in at least 30 cells. ** *p* < 0.01 when compared with PBS group, *n* ≥ 4; ^#^ *p* < 0.05 when compared with the LPS group, *n* ≥ 4.

Sepsis was the systemic inflammatory responses to infections, and the circulating macrophages played an important role during these responses through the release of inflammatory cytokines, such as IL-6 and TNF-α. Considering macrophages were the most typical cells in systemic inflammation, we isolated the macrophages from mice peritoneal cavity for the subsequent studies. LPS increased the levels of IL-6 and TNF-α in PM culture; whereas SIN-HCl decreased the release of IL-6 and TNF-α induced by LPS (Figure 4A). Meanwhile, autophagic activities were determined via autophagosome formation assay, and shown as LC3 puncta with green immunofluorescence. The autophagosome formation increased significantly in LPS or SIN-HCl treated PM, and the simultaneous treatment further increased the autophagosome formation, indicating that SIN-HCl could activate autophagy *in vitro* (Figure 4B).

### 2.4. The Effects of 3-MA on SIN-HCl-Induced Autophagy and Inflammatory Responses in PM

**Figure 5:**
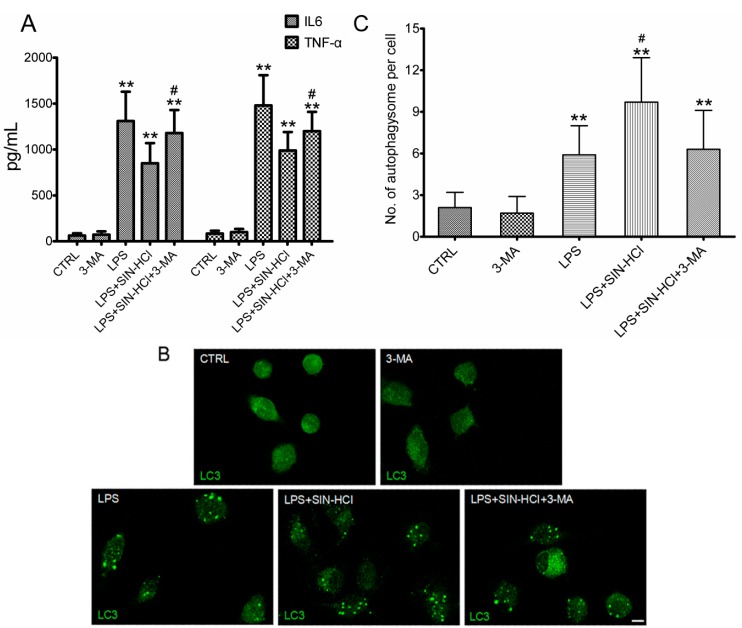
The effects of 3-MA on SIN-HCl-induced autophagy and inflammatory responses in PM. (**A**) Primary PM were cultured for 12 h with 3-MA (5 mM), LPS (100 ng/mL), SIN-HCl (100 mM), or a combination of these two reagents (as indicated). The levels of IL-6 and TNF-α in culture media were measured. ** *p* < 0.01 when compared with PBS group, *n* ≥ 6; ^#^ *p* < 0.05 when compared with the LPS group, *n* ≥ 6; (**B**) Primary PM were treated with 3-MA (5 mM), LPS (100 ng/mL), SIN-HCl (100 mM), or a combination of these reagents (as indicated) for 12 h before immunofluorescent detection of LC3 protein using an anti-LC3 antibody. Scale bar = 10 μm; (**C**) Autophagosomes in at least 30 PM cells were counted. Scale bar was equivalent to 10 μm. ** *p* < 0.01 when compared with PBS group, *n* ≥ 4; ^#^ *p* < 0.05 when compared with the LPS group, *n* ≥ 4.

To confirm whether the protective effects of SIN-HCl were involved in attenuating the release of inflammatory cytokines by regulating autophagic activities, we used autophagy inhibitor 3-MA to block autophagic activities and observed its effectiveness on SIN-HCl-induced autophagy and inflammatory responses. 3-MA treatment aggravated LPS-induced inflammatory responses and the protective effects of SIN-HCL were partially constrained (Figure 5A). Meanwhile, immunofluorescence results showed that SIN-HCl-induced autophagic activities could be inhibited by 3-MA (Figure 5B).

## 3. Discussion

In the present study, we demonstrated the protective effect of SIN-HCl against sepsis-induced mortality using a BALB/c mouse model of CLP and PM. Our findings focused on the potential of SIN-HCl as a new therapeutic option for sepsis.

We proposed that SIN-HCl might play a protective role in sepsis according to its anti-inflammatory and immunoregulatory properties. In order to test this hypothesis, we used a CLP mouse model, which was reliable and clinically relevant to the sepsis. The CLP procedure was commonly applied because it produced an endogenous polymicrobial infection that was similar to peritonitis and sepsis in clinic. Studies showed that CLP-induced septic mortality was significantly reduced in mice if they were immediately treated with SIN-HCl, indicating that SIN-HCl played a protective role in CLP-induced sepsis. This finding was consistent with previous study that SIN could attenuate endotoxemia [20]. Additionally, post-administration of SIN-HCl significantly improved the survival rates of mice, further indicating the potential of SIN-HCl as a new therapeutic option for sepsis. The decrease in organ damage and inflammatory cytokine production by SIN-HCl treatment also supported our hypothesis.

Clinical and laboratory-based studies have suggested that autophagy is activated during sepsis and involved in sepsis pathophysiology [6,21]. However, the role of autophagy in sepsis was not yet well defined. The majority of reports have shown that autophagy has a positive role in sepsis. For instance, macrophages deficient in the autophagy protein Atg16L1 could produce inflammatory cytokines after LPS stimulation [22], suggesting that autophagy plays an important role in regulation of endotoxemia. Polymorphism of autophagy-related genes (ATG16L1 and IRGM) was associated with severity and mortality of sepsis [23,24]. To clarify the relationship between SIN-HCl and autophagy in sepsis, we first determined the autophagy status in CLP-treated mice, and found that the ratios of LC3-II to LC3-I significantly increased in the liver, lungs, and kidneys of CLP-treated mice. SIN-HCl further increased the ratios of LC3-II to LC3-I in the liver and lungs, but had little effect on that in the kidneys. Then, we used LPS-treated PM in our *in vitro* study. Data showed that both SIN-HCl and LPS significantly increased the number of autophagosomes in individual cells when compared with the control group, and a combination of the two further increased the cellular autophagosome number, suggesting that SIN-HCl could activate autophagy *in vivo* and *in vitro*. Meanwhile, LPS-induced inflammatory cytokines were significantly reduced in the presence of SIN-HCl. Therefore, we speculated that SIN-HCl attenuated the release of inflammatory cytokines in CLP-treated mice and LPS-stimulated PM through regulating autophagy activities.

We further treated PM with autophagy inhibitor 3-MA, which inhibited class I and class III PtdIns 3-kinase and therefore would result in autophagy inhibition through suppression of class III PtdIns 3-kinase [25]. We found that 3-MA significantly decreased the numbers of LPS- or SIN-HCl-induced cellular autophagosomes. Meanwhile, decreases in inflammatory cytokines IL-6 and TNF-α caused by SIN-HCl were aggravated with 3-MA treatment. However, the protective role of SIN-HCl could not be eliminated completely by 3-MA. We postulated that other signal pathways were involved in the process.

In sepsis, autophagic processes were activated as protective responses to antagonize the injuries induced by sepsis even though they were less efficient. SIN-HCl further increased autophagy, to alleviate sepsis. However, there were some limitations to our study. On the one hand, we could not identify the direct relationship between autophagy and SIN-HCl in sepsis, due to the lack of effective intervention in mice. Our plan for a future study is to generate autophagy-related gene knockout or gene silencing in mice through virus mediation, and explore how SIN-HCl activates autophagy in sepsis. On the other hand, the immune system should play an important role in the pathophysiological processes of sepsis, and immune responses and processes were regulated by autophagy as well [26,27]. Next, we plan to do some studies to explore whether SIN-HCl and autophagy regulate the immune response in sepsis.

## 4. Experimental Section

### 4.1. Materials

Injectable SIN-HCl (Zhengqing Fengtongning injection) was purchased from Zhengqing Pharmacy Co., Ltd. (Changsha, China). Endotoxin was not detected in this SIN-HCl solution using ToxinSensor™ Chromogenic LAL Endotoxin Assay (GenScript, Nanjing, China) (date not shown). SIN-HCl was dissolved and diluted using sterile normal saline in all *in vitro* and *in vivo* experiments.

### 4.2. Animals

Male BALB/c mice (6–8 weeks old) were provided by SJA Laboratory Animal Co., Ltd. (Changsha, China). The mice were allowed to acclimate to specific pathogen-free conditions with food and water supply on 12:12 h day/night cycle for at least 7 days before experiments. All procedures performed were approved by the Institutional Animal Ethics Committee and conformed to the Guidelines of Laboratory Animal Care and Use Committee at the Xiangya School of Medicine, Central South University (Changsha, China).

### 4.3. CLP Model and SIN-HCl Treatment

CLP was performed as described previously [28]. First, experimental mice (6–8 weeks old) were anesthetized with 5% chloral hydrate (300 mg/kg of body weight) and laid on the operating table. A 20 mm midline incision was made to expose the cecum. The cecum was ligated below the ileocecal valve without causing intestinal obstruction, and then the ligated cecum was punctured twice with a 21-gauge needle. Finally, cecum was replaced in its normal intra-abdominal position and the wound was closed with a running suture. In the sham-operated mice, the cecum was exposed as described above; however, they were not ligated or punctured. After surgery, the mice were injected subcutaneously with 1 mL SIN-HCl or sterile NS solution for fluid resuscitation. Morphine were injected 0.1 mg/kg subcutaneous injection (s.c.) for postoperative analgesia. Repeat every 6 h for 48 h. At the indicated time, mice were anesthetized and tissue and blood samples were taken for further analysis. Then the mice were euthanized. Survival was evaluated for 7 consecutive days after CLP.

### 4.4. Serum Biochemical Parameters and Cytokine Determination

At 24 h after the surgery, mice were anesthetized and blood was drawn from the heart; then mice were euthanized and blood was centrifuged at 3000× *g* for 15 min at 4 °C. Fresh serum samples were analyzed to assess the biochemical parameters [blood urine nitrogen (BUN), creatinine (Cr), alanine transaminase (ALT), and aspartate Transaminase (AST)] using commercially available clinical assay kits on an Olympus AU5400 Automatic Biochemical Analyzer (Olympus, Tokyo, Japan). The serum leftovers were stored at −80 °C prior to enzyme-linked immunosorbent assay (ELISA) analysis. The results were expressed as mean ± SD of six to eight separate samples. Interleukin 6 (IL-6) and Tumor necrosis factor α (TNF-α) in the serum and culture medium were measured using ELISA (the kits were purchased from Boster Biological Technology, Wuhan, China) following the manufacturer’s recommendations. The results were expressed as mean ± SD of six to eight separate samples.

### 4.5. Morphological Analysis

Morphological analyses were performed as previously described [29]. The lung, liver, and kidney tissues were fixed in 4% paraformaldehyde for 24 h. The samples were dehydrated through increasing concentrations of ethanol (50%–100%), and then placed in xylene for 3 h, followed by overnight paraffin embedding. Sections (4 μm in thickness) were prepared and mounted on slides. The slides were placed in xylene, followed by deparaffinization through decreasing concentrations of ethanol (100%–50%). The slides were stained with hematoxylin and eosin (HE) and examined under a light microscope. Histopathological injuries of lungs were evaluated semi-quantitatively using Smith’s method [30]. Briefly, infiltration of inflammatory cells, interstitial edema, congestion, hemorrhage, hyaline membrane formation, and necrosis were each scored on a scale of 0–4: 0, normal; 1, minimal (<25%); 2, mild (25%–50%); 3, moderate (50%–75%); and 4, severe (>75%). Hepatic injury scores were obtained by measuring hepatocellular necrosis, hemorrhage, hepatic parenchymal inflammatory infiltrate, and sinusoidal inflammatory infiltrate according to the morphologic criteria previously described by Coimbra *et al.* [31]. All scores also ranged between 0 (normal) and 4 (severe). Structural changes in kidney tissue sections were evaluated as previously described by Yasuda *et al.* [32]. Proximal tubule damages, inflammatory cells’ infiltration, hemorrhage, interstitial structural changes, renal corpuscle morphology, and necrotic cells were scored semi-quantitatively on a scale of 0–4: 0, none; 1, <25%; 2, 25%–50%; 3, 50%–75%; and 4, 75%–100%. Tissue sections were examined by an experienced pathologist who was kept blind about the study designs.

### 4.6. Western Blotting Analyses

Western blotting was performed as previously described [33]. The lungs, liver, and kidneys were rinsed twice with ice-cold PBS and incubated with RIPA lysis buffer (50 mM Tris, 150 mM NaCl, 1% Triton X-100, 1% sodium deoxycholate, 0.1% SDS, pH 7.4, Beyotime (Nantong, China)) containing a protease inhibitor phenylmethanesulfonyl fluoride (PMSF, 1 mM). Protein extracts were centrifuged at 13,000× *g* for 5 min, and the supernatants were quantified by BCA protein assay (Beyotime). Total proteins (20–30 mg per lane) were separated on a 15% SDS/polyacrylamide gel (SDS-PAGE) and transferred to polyvinylidene fluoride membranes (PVDF, Millipore, MA, USA). The membranes were blocked with 5% bovine serum albumin in TBS-T (20 mM Tris, 150 mM NaCl, pH 7.5 containing 0.1% Tween-20) for 2 h at room temperature, followed by incubation with a rabbit polyclonal antibody against LC3 (1:1000; MBL, Nagoya, Japan) or a mouse monoclonal antibody against β-actin (1:1000; Sigma-Aldrich, St. Louis, MO, USA) at 4 °C overnight. The membranes were rinsed three times (15 min each) with TBS-T and then incubated with an appropriate horseradish peroxidase-conjugated IgG secondary antibody (diluted 1:2000 in TBS-T buffer) for 1 h at room temperature. Finally, the membranes were rinsed three times (15 min each) with TBS-T and incubated with DAB reagent according to the manufacturer’s instructions (Boster Biological Technology, Wuhan, China). The protein expression levels were quantitatively analyzed using ImageJ software (NIH, Bethesda, MD, USA) and normalized against β-actin loading control.

### 4.7. Isolation of Peritoneal Macrophages and Cell Treatments

The murine PMs were isolated and purified as described previously [34]. Mice were injected intraperitoneal injection (i.p.) with 3 mL thioglycolate broth (Sigma-Aldrich); and three days later, PMs were recovered by peritoneal lavage with Dulbecco phosphate-buffered saline. Macrophages were further purified by adherence to culture dishes for 7 days. The purified macrophages were treated with 100 ng/mL LPS (Sigma-Aldrich), 5 mM 3-MA (Sigma-Aldrich), and 100 μM SIN-HCl, respectively, for 12 h.

### 4.8. Immunofluorescence Staining

PMs on cover glass were washed with PBS three times and fixed in 4% paraformaldehyde. After blocking in PBS with 10% fetal bovine serum for 30 min, the fixed cells were incubated with primary antibody LC3 (1:1000, MBL), followed by incubation with secondary Alexa-Fluor 488-conjugated donkey antibody to rabbit IgG (1:1000, Invitrogen, Waltham, MA, USA). Cells were then washed with PBS three times and imaged using a confocal microscope (Leica Camera AG, Solms, Germany). At least 30 cells were examined.

### 4.9. Statistical Analyses

The quantitative data were presented as means ± SD, and one-way ANOVA combined with Student-Newman-Keuls or Dunnett’s tests were used to compare the differences between groups. Mortality comparisons were performed by Kaplan-Meier survival curve analysis. The logrank test was used to assess survival differences. A value of *p* < 0.05 was considered statistically significant.

## 5. Conclusions

Taken together, our results indicated that SIN-HCl could improve survival, reduce organ damage, and attenuate the release of inflammatory cytokines induced by CLP, at least partially through regulating autophagy activity. Our data suggested that SIN-HCl might present a new therapeutic option for sepsis through modulation of autophagy.

## Supplementary Materials

Supplementary materials can be found at http://www.mdpi.com/1422-0067/16/02/2559/s1.

## Abbreviation

SIN: Sinomenine
SIN-HCl: Sinomenine hydrochloride
CLP: Cecal ligation and puncture
PM: Peritoneal macrophages
LPS: Lipopolysaccharide
LC3-II: Microtubule-associated protein light chain 3-II
LC3-I: Microtubule-associated protein 1 light chain-3-I
TNF-α: Tumor necrosis factor α
IL-6: Interleukin 6
3-MA: 3-Methyladenine
ICU: Intensive care unit
ELISA: Enzyme-linked immunosorbent assay
